# Non-Indigenous women’s experiences of obstetric and gynaecological inequity in rural and remote South Australia: A feminist post-structuralist analysis

**DOI:** 10.1177/17455057261444883

**Published:** 2026-06-09

**Authors:** Laura Nolan, Elizabeth Newnham, Jacqueline H Stephens

**Affiliations:** 1 Flinders Health and Medical Research Institute, College of Medicine and Public Health, Flinders University, Adelaide, SA, Australia; 2 College of Health and Enablement, Flinders University, Adelaide, SA, Australia

**Keywords:** women’s health, reproductive health, decision-making, health inequities, rural health services

## Abstract

**Background:**

Women and people with uteruses (“women”) living in rural and remote areas face inequitable access to obstetric and gynaecological (OB-GYN) health services.

**Objectives:**

This research aimed to understand how women experience and navigate access to OB-GYN health care in rural and remote settings.

**Design:**

A phenomenological design was utilised to examine women’s lived experiences of OB-GYN services. To cultivate a critical feminist standpoint, the researchers then applied a feminist post-structuralist framework, which reflexively attends to the interplay of power, discourse, gender, and geography in lived experience.

**Methods:**

Semi-structured interviews were conducted with 14 women living in rural and remote South Australia between September 2022 and January 2023. An iterative, open-coding, thematic analysis was chosen by researchers to synthesise the findings whilst retaining the richness of the data.

**Results:**

Four themes were identified. First, access was difficult due to systemic power imbalances. Second, continuity of care was built upon intersubjectivity. Third, health literacy was key to informed consent, choice, and control. Last, shared knowledge translation redefined OB-GYN health services.

**Conclusion:**

The systematic othering of women and inequitable rural resourcing together produce health and power inequities for rural South Australians with uteruses, denying their experiences and bodily autonomy.

## Introduction

Obstetric and gynaecological (OB-GYN) health services are a key component of the health and agency of women and people with uteruses (henceforth referred to as “women”), irrespective of their pregnancy intentions. Historically, such services have narrowly been shaped by an andro-centric, bio-medical prioritisation of pregnancy and fertility health.^1^ A life-course perspective, however, brings attention to OB-GYN services intertwinement with a person’s socio-psychological and physical health,^1^ inclusive to reproductive agency,^2–4^ cancer screenings,^5^ pelvic pain treatment,^6^ informed decision-making,^7^ mental health services,^8^ and domestic violence support.^9^ Access to choices regarding these services is an essential part of OB-GYN health care,^10^ however, largely entangled with systemic inequities.^11^

Worldwide, ongoing processes of colonisation systemically exclude First Nations women’s equitable access to, and outcomes of, OB-GYN health care and services.^12^ Aboriginal women living in the Far West Coast of South Australia were included in this study. To deeply listen to, and learn from, the intersectionality of Aboriginal women’s experiences with a decolonial lens, their yarns are reported separately to this article.

Application of intersectionality, a critical feminist paradigm, attends to how social identities, such as gender, class, and race, intersect, and therefore, give rise to co-constitutive and distinctive experiences of disparity and privilege.^13,14^ Herein, this paradigm is critical due to its capacity to analyse beyond a single axis of gender, and instead, uncover entangled systems of power that sculpt and reinforce inequity.^13,14^ This lens illuminates how OB-GYN services are enmeshed with social identities and structures, manifesting into real-world services as well as broader social determinants of health.^11^ For instance, gender intersects with geographical remoteness and poverty on a global scale,^15^ contributing unevenly to approximately over 200 million women not having access to contraceptives,^13,14^ mortality from breast cancer (670,000 deaths),^16^ as well as 90% of maternal deaths.^15^ All of these unevenly effect women living in low- and middle-income countries, most significantly in rural and remote areas.^17,18^ These intersecting gendered, geographical, and health disparities persist, despite advancements in modern OB-GYN medicine, as seen in the most treatable form of cancer being gynaecological cancer, yet such forms of cancer continue to cause over 600,000 deaths.^17,19^

Despite Australia’s health system ranking highly amongst OECD countries,^20^ systemic gender bias is increasingly reported in Australian healthcare services.^21^ One in 10 Australian women experience obstetric violence, which includes bullying, disrespectful language, and coercion during childbirth, increasing their risk to postnatal depression and post-traumatic stress disorder (PTSD).^22,23^ Faced with geographical remoteness and limited local health services, women living in rural and remote Australia experience poorer health outcomes and inequities than their urban counterparts.^24^ Travelling far distances to access women’s health services exacerbates out-of-pocket expenses, logistical hardships, and socio-economic independence.^25^ Barriers for women living rural and remotely are entangled with lower socio-economic backgrounds and higher risks of experiencing domestic violence.^24^ Women’s exertion of agency, however, is critical to their access to OB-GYN health care,^26,27^ yet exercised within these uneven, systemic conditions and relations of power.

To address such inequities, a feminist post-structuralist framework applies an intersectional lens to reflexively attend to how gender intersects with living in rural and remote areas, forming distinctive subjective experiences of OB-GYN services. Previous literature has used this framework to examine how women’s health choices are influenced by their “experiential wisdom, their lived and bodily experiences, and knowledge.”^28^ Much of the existing literature largely attends to OB-GYN health care as distinctive experiences, with studies independently examining unintended pregnancies,^29^ maternity services,^30^ or cervical cancer screenings^31^ and thus neglecting their interconnectedness to women’s embodied knowledge and health.^32^ By phenomenologically exploring women’s subjectivity, their embodied and situated knowledge offers understanding of complex social and systemic power relations, which are navigated alongside health literacy,^33^ informed consent,^30^ pain management,^32^ and unsafe healthcare settings. In foregrounding women’s experiences, a feminist post-structuralist lens subverts dominant bio-medical discourses,^23^ and recenter women’s agency in accessing OB-GYN services.^28^ Despite use of critical feminist paradigms expanding in Australian research, fewer studies have applied this lens in rural and remote contexts of OB-GYN health services. Yet, this paucity of research warrants timely consideration, as women living in rural and remote areas are classified as a priority health population in Australia.^34^

Herein, this article aimed to apply an embodied, women-centred approach to understanding rural and remote non-Indigenous women’s subjective experiences of OB-GYN health care.^9^ Two research questions were developed to guide this study, asking: (1) how do women in rural and remote South Australia townships navigate OB-GYN health care and (2) how these women conceptualise OB-GYN health care? By applying a feminist post-structuralist framework to our analysis, we believe this is the first qualitative explorative study to utilise an embodied, feminist, approach to understand non-Indigenous women’s subjective experiences of OB-GYN health care in rural and remote South Australia.

## Methods

We undertook a qualitative cross-sectional study, conducting semi-structured interviews with non-Indigenous women living in rural and remote South Australia between September 2022 and January 2023. We report the findings in accordance with the Standard for Reporting Qualitative Research guidelines (Supplemental Material).^35^

### Theoretical framework

A feminist perspective was deemed necessary to examine the intersectionality of gender, geography, and class which influences women’s health experiences in rural and remote Australia.^28,36^ A post-structuralist approach was deemed necessary to expose, and thereby dismantle, domineering patriarchal and city-centric rhetorics that intersect and are entangled in bio-medical knowledge systems. Drawing upon Alspaugh et al.’s feminist post-structuralist framework, we critically explore how gender and geography uniquely intersect in OB-GYN services, re-centring women’s embodied experiences, power, and knowledge in negotiating health care in rural and remote South Australia. Crucially, it examines intersecting gendered and geographical experiences and inequities that vary for women living rurally or remotely. Using the five principles of this framework – discourse (knowledge), power, language, subjectivity and agency – we acknowledge each women’s reality is constructed by discourse, which produces knowledge and a system of meaning through language, thus placing their subjectivity within complex power relations.^37,38^ This framework recognises that power relations operate beyond male-dominated relationships, and discourses are redefined by women’s own knowledge, choices, and agency.^28^ Determinants of agency are shaped by women’s access to higher education and decision-making skills.^39^ This feminist post-structuralist framework was applied during data analysis and supports the reflexive examination of knowledge production within academic research and health practices.

### Qualitative approach and research paradigm

Reflective of our feminist post-structuralist framework, this research study was undertaken with a critical positioning to explore participants situated knowledge by de-constructing hegemonic discourses. To achieve this, reflexive thematic analysis was utilised to analyse the data. This approach understands the researchers’ subjectivity is an instrument in the research process, rather than hindrance, with researchers’ knowledge implicitly situated rather than objective.^40^ Researchers reflected on being cis-women, having access to city-based services, their socio-economic status, and experiences of patriarchal bio-medical discourses. Themes were therefore not pre-existing or discovered in the data, but rather the systematic analysis and development of themes was shaped by our own positioning and meta-theoretical perspectives.^40^

### Researcher characteristics and reflexivity

The researchers are women. The senior author (JS) is an epidemiologist with a background in community-based mixed-methods research in rural and remote locations. The first author (LN) is an early-career researcher with experience in conducting qualitative and reflexive research methodologies. EN is a midwife and academic with a background in critical qualitative research methodologies. The authors have diverse experience across public health disciplines and social sciences. Throughout data collection and analysis, two researchers (JS and LN) iteratively engaged in reflexivity to acknowledge their own subjectivity in understanding and interpreting the data.^40^ Rather than preventing a subjective evaluation, applying a reflexive lens allows researchers to acknowledge how their own subjectivity is entangled in identifying research themes. To minimise the assumption of whiteness as universal and invisible, researchers reflexively acknowledged during data collection and analysis that all participants in this study had a European-Anglo background and had experiences positioned on an axis of privilege within the Australian context.^41^ Furthermore, throughout the study researchers reflected on their own experiences of women’s health and health care, which was critical to understanding the data beyond traditional medical understandings.

### Context and setting

Data collection took place in two locations in South Australia: Ceduna and the Riverland. There is a vast 820 km between these two locations, with both providing enmeshed, yet distinct, examples of power dynamics that intersect between women, as healthcare seekers, and their OB-GYN health services, as systems of power. In both locations, women’s experiences are influenced by the intersectionality of their gender, geography, class, and privilege as white woman. Yet, in comparison with the Riverland, women living in Ceduna are disproportionately affected by their intersecting geographical remoteness to city-centric services. The Modified Monash Model and Australian Statistical Geography Standard Remoteness Areas were used to determine remoteness; both were consistent with Ceduna considered remote, and the Renmark/Riverland region considered rural.^42,43^

Ceduna is a remote coastal township on the far west coast of South Australia, located 780 km (8 h) from the state’s capital city and home to approximately 2000 people. The town is served by one state government–funded (public) hospital, several general practices, and an Aboriginal Community Controlled Health Service.

The Riverland is a rural agricultural region covering 9400 km^2^, which is home to approximately 35,000 people. The region consists of five main towns situated along the Murray River, with the main town (Renmark) located 260 km^2^ (3.5 h) from the state’s capital city. The region is served by a comprehensive network of hospitals, general practices, and community-based health services.

### Ethical considerations

Ethics approval was provided by the Aboriginal Health Research Ethics Committee (App 04-22-981) and the Flinders University Human Research Ethics Committee (App 5543). This study was conducted as part of a larger body of work in which Aboriginal women living in the Far West Coast region were also interviewed about their experiences of women’s health care. For ethical and cultural reasons, Aboriginal women’s experiences were analysed separately and will be reported elsewhere. Knowledge co-produced from the interviews with non-Indigenous women, as reported herein, is acknowledged to belong to the women involved in this research. For reporting purposes, each participant has been allocated a pseudonym to protect their identities. Similarly, where specific hospitals, doctors, or healthcare services were named by women during the interviews, these have been allocated a non-identifying descriptor.

### Study population

Women were eligible to participate if they were aged over 18 years, did not identify as Aboriginal and Torres Strait Islander, and had used obstetric and/or gynaecological services in the past 12 months. There were no exclusion criteria.

### Participant recruitment

The lead researcher (JS) used existing community connections for recruitment. Convenience sampling was undertaken to recruit participants at local health clinics, community events, and parenting groups across the two study locations. Posters with a QR code to an online registration form were displayed in participating clinics. Social media (e.g., Facebook) was used to distribute the posters online as free and/or paid targeted adverts and posted to Facebook groups specific to the two regions. Women who registered interested were contacted via e-mail or telephone, depending on their preference, and provided with more information about the purpose of the study. Prior to this study, the senior researcher (JS) did not have an established relationship with any participant.

### Data collection

Verbal consent was reaffirmed at interview commencement. Interviews were conducted in-person, at a time and location convenient to the participants, with some participants being interviewed in small groups of two to three women, for example, mother–daughter pair. To ensure confidentiality and to create a safe space for participants, men (such as partners, husbands) were not present during interviews. A predefined interview guide was developed and used (Supplemental Material). The interview guide was validated through reflexivity, expert review, and peer debriefing to review questions for content validity. However, whilst it was used as a guide, the semi-structured interviews employed an informal, organic approach to the conversations with women able to focus on topics of importance to them and thus prioritising their experiences of what is most influential to their health care. All interviews were conducted by the senior author (JS) with post-interview reflexive analysis and note-taking conducted immediately after the sessions. The predictability and consistency of insights and topics were reviewed and when new interviews no longer provided additional insights or understandings, meaning saturation was deemed to be achieved. All interviews were audio-recorded, transcribed, and deidentified by an independent transcription service before analysis.

### Data analysis

Initial-open and axial coding was undertaken by the first author (LN), according to Braun and Clarke’s thematic analysis framework. Identification of codes in the transcripts was conducted whilst listening to the interview audio files to ensure immersion in the data. This allowed codes and evolving themes to directly correspond with participants lived experiences.^40^ Data from women living in rural locations were analysed separately from those living in remote locations; however, both were open-coded to identify all potential codes in the data. Once open coding was complete, identification of similarities and differences between the experiences of the two groups of women were identified. Consensus discussions occurred regularly during the open-coding stage to strengthen reflexivity. The open- and axial-coding phases were inductive, leading the first and senior authors (LN and JS) to identify tension between women’s subjective knowledge and patriarchal, bio-medical discourses. This led to a search for a guiding theoretical lens, with the feminist post-structuralist framework being identified and integrated into the data analysis. The selective coding phase was then deductively guided by the framework, whereby we mapped the inductively identified codes to the five concepts of the framework to understand how the tension between knowledge, discourse, and power correlated with women’s subjective experiences of OB-GYN health care.^28^ The data management of this iterative coding and thematic analysis process was performed in the NVivo software (Lumivero, Release 1.7) by the first author (LN). Following the identification of the key themes and core relationships, a thematic map was constructed using graphic software (Canva.com), offering a visual exploration of how themes and sub-themes interrelated.

## Results

Thirty women aged over 18 years and residing in the 2 study locations expressed interest in participating in the study, with 17 signing written consent to participate. Of these, 15 women participated in face-to-face semi-structured interviews between 12 September 2022 and 5 January 2023. Seven (46.7%) women were from Ceduna; the remainder were from the Riverland region. The women’s median age was 30 years old (range: 19–53). Most participating women were in their late 20s to early 30s, with one being in her 50s with an adult daughter also in the study. Interviews ranged in length from 54 to 93 min. All participants identified as non-Indigenous, from European “white” backgrounds. A feminist post-structuralist paradigm, with an intersectional lens, is critical in this sense, as it attends to the intersectionality of race, specifically in Australian context, where colonial power relations intersect with dominant discourses, reaffirming the invisibility, and thus privilege, of whiteness. Thus, women in our study are entangled in an axis of racial privilege, which intersects with their gendered and geographical inequity, and distinctively shapes their embodied experiences of health.^13^ This privilege must be unsilenced to expose intersecting systemic inequities, which disproportionately impact black, Indigenous, and women of colour (BIPOC), and to curate critical health policy and praxis.

### Themes and thematic map

**Figure 1.**
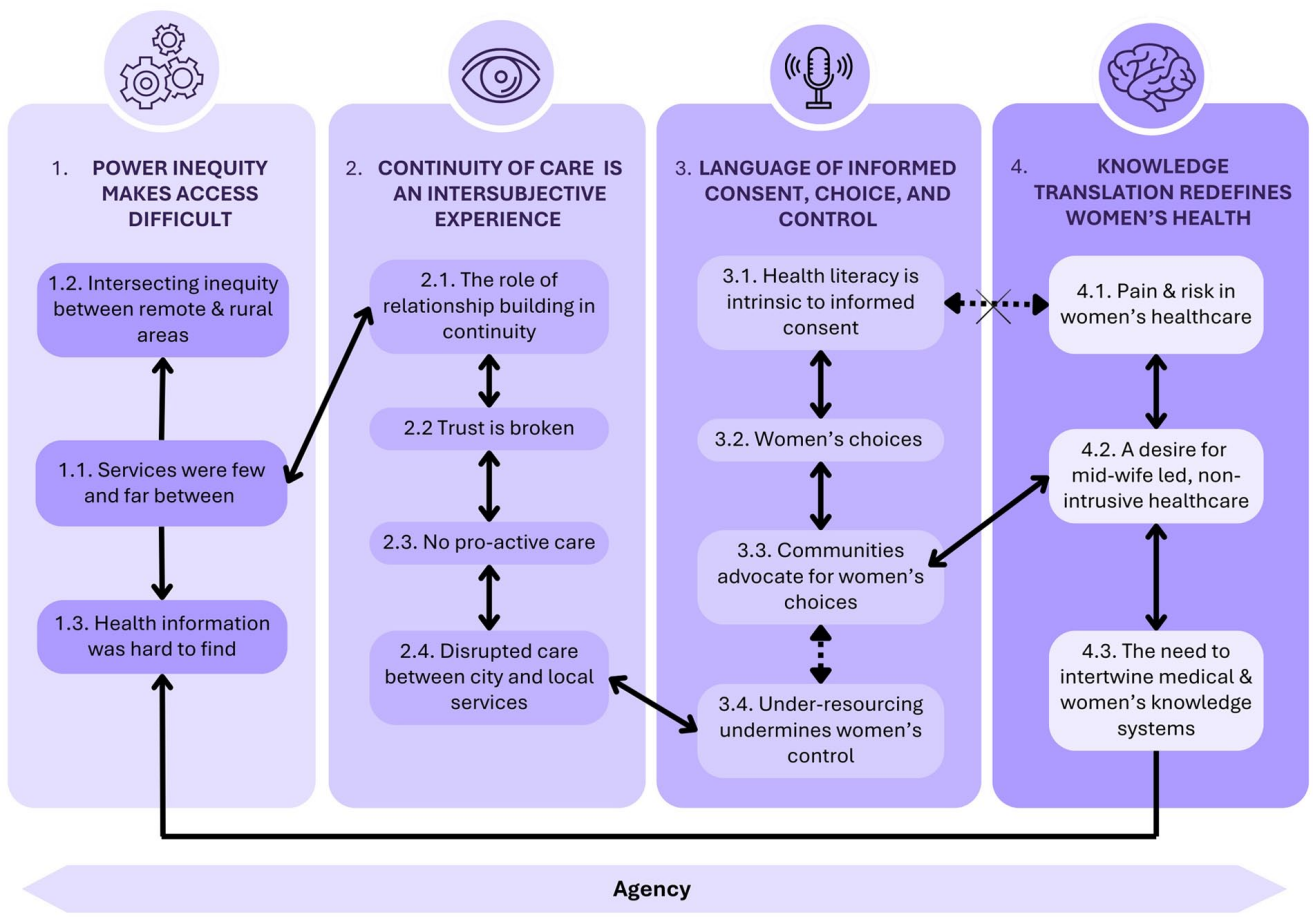
Thematic map exploring participants’ experiences of women’s health care in rural and remote South Australia.

A two-step inductive and deductive analytical approach was followed. First, during inductive analysis, we established that insufficient access, continuity, choice and consent, and traumatic health care settings were widespread in women’s experiences. At the same time, however, many women identified that these experiences were systemically entangled with gender and geographical inequity. This led researchers to search for, and apply, a critical feminist post-structuralist paradigm, with the intention to examine the underlying power imbalances, discourses, and knowledge regimes that reproduce such inequity. During this second step, we mapped the inductively identified codes to the feminist post-structuralist framework. During this deductive analysis, four themes were constructed, as follows: (1) power inequity makes access difficult; (2) continuity of care is an intersubjective experience; (3) the language of informed consent, choice and control; and (4) knowledge translation redefines women’s health. The thematic map (Figure 1) illustrates how these themes intersect with the lived experiences of participants. The four themes deductively align to the principles of a feminist post-structuralist framework: power, subjectivity, language, and knowledge, with agency being enacted, negotiated, and mediated across all four themes.

### Theme 1: Power inequity makes access difficult

Power inequity emerges through the intersecting effects of geography and gender, shaping how women in rural and remote areas experience and navigate OB-GYN health care. Subthemes 1.1 and 1.2 describe how city-centric systems distribute services in ways that marginalise rural women and prioritise urban biomedical authority over local autonomy. Subtheme 1.3 highlights how limited access to information restricts women’s knowledge, undermining their agency and informed decision-making.

#### Subtheme 1.1: Services were few and far between

In both rural and remote locations, women drew links between feeling powerless to access health services, and a shortage of local and female health professionals. Women delayed accessing cancer-screenings due to the absence of a female doctor.

“I never wanted a pap smear done by any of the other male doctors that are here; just don’t like them, don’t trust them. If I had to, I would have just waited for a female locum.” (Faye, Ceduna)

Women believed local health professionals were burnt-out, leading to a “rolling issue in getting [health professionals] to come to the country and stay in the country” (Cate, Ceduna). Women reported a need for government funded schemes to reduce university debts for graduates working long-term in rural and remote communities, enabling them to “stay grounded in their community” (Jane, Riverland).

Scarcity of local resources led to women waiting up to 6 months for medical appointments. Milestone pap-smear and obstetric appointments were missed due to clinics being overbooked: “you have to be willing to wait” (Diana, Ceduna).

“Oh, [doctor’s] sick today. . .so your two months’ appointment you’ve been waiting for has been cancelled, so you may have to wait another two months.” (Hana, Riverland)

Neither community had a local gynaecologist or lactation consultant. Tele-health appointments were available but are not always appropriate for intimate women’s health checks. Women reported insufficient clinical psychologists or specialist doctors in postnatal depression, with one woman stating: “mental health is really hard in the [Riverland] because there are no doctors available” (Kira, Riverland). Contingency planning trips to Adelaide, or other regional towns, was always necessary for women to consider. The financial inequity of rural health care was recalled by women who travelled earlier in their pregnancy to access regional and city-based services, which resulted in disproportionate out of pocket costs compared to their urban counterparts, amassing through the logistical expenses of petrol, groceries, accommodation, as well as irregularities to their household’s employment and income. Women diagnosed as having a “high risk pregnancy,” such as a body mass index over 40, were required to travel to Adelaide at the 36th week to be near a tertiary care hospital with appropriate facilities, amassing financial costs.

#### Subtheme 1.2: Inequity between remote and rural areas

The intersectionality of women’s experiences according to their degree of remoteness or rurality was evident, and was intertwined with their socio-economic class, age, and healthcare needs.

##### Accessing remote health care: Ceduna

Ceduna’s geographically isolated location reduced women’s access to health care, whilst intersecting with women’s financial capital, age, class, and employment. With 800 km to Adelaide, women recounted that if something goes wrong “you’re stuck and that’s where it gets scary” (Anne, Ceduna). Despite Port Lincon being the closest regional city, the long drive required women to travel “four hours down, four hours back” (Emma, Ceduna), incurring travel-related out of pocket expenses and inequity, often whilst experiencing severe pain or unstable health conditions. One woman recalled travelling with her newborn baby, who required an oxygen tube to breath:

“We drove back with an oxygen tank, and we had to have overnight freight or a BOC dealer, so they would swap our tanks over, and then we had all the equipment come for the hospital.” (Faye, Ceduna).

Severe under-resourcing resulted in periods with no available midwives, obstetricians, theatre nurses, or anaesthetists; “if something had happened there would have been no one here” (Gina, Ceduna). Birthing induction dates were rescheduled, or suspended, due to staff shortages; “I got given four different induction dates that they kept – they cancelled last minute” (Diana, Ceduna). Women recalled being forced to relocate their pregnancy and birthing care to Port Lincon, Port-Augusta, or Adelaide, prior to being 36-week pregnant. With their families accompanying them, women recalled re-location negatively affecting their income.

Women reported there was a waiting period of up to 6 weeks for the residential obstetrician. Government-run sexual health service no longer visited, and a government-employed child health nurse only visited the town once every 6 weeks.

Travel assistant schemes only supported transport to pap-smear screenings that were geographically the closest service, being Whyalla (a 4-h drive), rather than supporting the quickest access, being flights to Adelaide. Women recalled how this negatively impacted older women’s access when not confident drivers. However, local Aboriginal Medical Services were often accessed by non-Indigenous women, enabling timely, inexpensive, and care provided by female doctors who specialised in women’s health. Women recalled having to organise their own way back to Ceduna; “they wanted to put me on the bus” (Faye, Ceduna).

##### Accessing rural health care: The Riverland

The Riverland is less geographically isolated, strengthening women’s access to health care, and minimising travel-related out-of-pocket costs. Women’s pregnancy care is offered at two hospitals (both locally within a 30-min drive) and a 3-h drive from Adelaide. Women living in the Riverland had daytrips to Adelaide for healthcare services; however, they found this negatively intersected with financial and social stresses:

“[W]e’d leave here at 4 o’clock in the morning, drive to Adelaide, I’d have my bloods and ultrasounds done, and then we’d drive back, my husband would drop me off at work at 12 o’clock when I started, and then I’d get home at 4 o’clock . . . that was hell” (Jane, Riverland).

Rather than travelling to Adelaide, women desired to access interstate healthcare services in Mildura. However, poor interstate and private-to-public data sharing was a barrier: “you can’t just get on the system” (Isla, Riverland).

Women in this study expressed annoyance that women’s health care was centralised at one health service: with one woman commenting that “you didn’t get a choice” (Isla, Riverland). Women were commonly placed with unsuitable doctors: “we couldn’t even see a baby doctor” (Hana, Riverland).

Riverland’s health services were expensive, and this deterred women from seeking appointments. Instead, some women attended the local emergency department to avoid paying out of pocket fees. For example, one woman stated: “I just need a little bit of reassurance on how my baby’s breathing” (Mila, Riverland). Accessing private lactation consultants and new patient fees were also cost prohibitive for women, for example:

“I don’t know what I would have done if we didn’t have the money because I probably just wouldn’t have been able to breastfeed.” (Lana, Riverland)

Services were determined, like Ceduna, to be unreliable and inconsistent. Women expressed frustration that fertility clinics were not located in their regions, the government-funded mammogram bus and locum radiology service visited irregularly, and the publicly funded physiotherapist had a 12-month waitlist; with one woman recalling: “I’ve got no hope having [ultrasounds] done at home” (Isla, Riverland)

#### Subtheme 1.3: Health information was hard to find

Health information was “not advertised or put out there” (Emma, Ceduna), causing women to miss the locum gynaecologists and mammogram services that were available. Women noted information was only spread if other women “say something or hear something in a conversation” (Emma, Ceduna). There was desire for public health education to strengthen health literacy within local communities. For example, two women stated:

“I didn’t even know to ask for it, obviously with my other pregnancies that had just been done. And that really scared me.” (Hana, Riverland)

“[W]hen I was first pregnant with [child’s name], I was like, what do you even do when you’re pregnant? Like what is the first port of call?” (Anne, Ceduna)

Another woman recounted not being able to find information about where her local Child and Family Health Service early parenting group was held, as described:

“[S]o turned up on their Tuesday drop in, opened the door, walked in, and I had a room full of builders staring at me.” (Isla, Riverland)

To overcome poor access to health resources, women noted the importance for continuity of care with health professionals, to connect to women’s individual understanding of her holistic health needs.

### Theme 2: Continuity of care is an intersubjective experience

Continuity of care was constructed as an intersubjective experience, where the relational space between a woman and her healthcare team shapes her subjectivity, that is, women’s understanding of their holistic health, body, and self. Subthemes 2.1 and 2.4 illustrate how consistent, trusting relationships foster a sense of support, whilst geographical disruptions caused by travel to city-based services fragment this shared understanding; whilst subthemes 2.2 and 2.3 highlight how broken trust and a lack of proactive care – often occurring when health professionals disregard women’s embodied experiences – undermine their agency in navigating a singular, often inflexible, regional health team.

#### Subtheme 2.1: The role of relationship building in continuity

Continuity in women relationship-building with their midwives and doctors was identified as crucial to “always [feeling] supported, listened to, heard” (Anne, Ceduna). Another woman stated about her midwife, “You could tell that she cared. She would message you just randomly just to check in.” (Emma, Ceduna)

However, workforce shortages across regional townships, particularly in Ceduna, led to women suddenly having broken relationships with their midwives and doctors, disrupting continuity. As one woman recalled her pregnancy care below:

“I saw the same midwife, same doctor my whole pregnancy. And then, the day I actually her, I didn’t have them. The doctor had flown out that morning. I was so angry because we had this plan. She’s the one that was supposed to deliver her. It was part of the deal why I birthed in Ceduna. So then when this other chick walked in, I’m like, who are you?” (Cate, Ceduna).

This was despite midwifery continuity of care models in both locations, whereby a woman is allocated a midwife dedicated to their pregnancy care until 6 weeks postpartum.^44^

Systemically, Riverland women noted continuity in relationship-building was embedded in their local maternity system, with one woman describing, “we had to see each GP obstetrician, so if one of them attended your birth, you’d know all of them.” (Hana, Renmark).

#### Subtheme 2.2: Trust is broken

Trust was repeatedly broken when health professionals did not share women’s understanding of their embodied health. Male, locum, and inexperienced health professionals recurrently did not listen to women’s lived experiences and “the relationship felt different” (Cate, Ceduna). Women in our study population actively sought female doctors, even if it delayed their access to health care. This was often due to previous negative experiences, as illustrated by the following quotes:

“[H]e asked me if I was sure that my baby’s dad was my baby’s dad. Because my dates didn’t match his. . .too bad if I had been a domestic violence [case].” (Faye, Ceduna)

“[H]e had no idea how to speak to women about their reproductive issues . . . didn’t seem to know how to deal with speaking to women in general . . . do your usual thing, and [he] just waved at the bed.” (Jane, Riverland)

When trust was broken, women recalled how this negatively impacted their well-being, as described by one woman with a graduate midwife:

“I was so nervous leading into labour, thinking, how much experience is this midwife actually going to have when it comes to delivering babies? . . .I was in early labour for a good week and a half of labour, starting and stopping nearly every night . . . I feel like my body was doing that because I knew that I didn’t feel comfortable working with this particular midwife.” (Emma, Ceduna)

Women found it difficult to book appointments with reception staff whilst maintaining confidentiality, and many women were not comfortable to explicitly book cervical screenings. When women lost trust in their health professionals, it was reported to be “very challenging because there’s only one team” (Anne, Ceduna). Shared understandings with female, local, and experienced health professionals improved women’s access to trustworthy health care. The following quotes illustrate these experiences:

“[T]he nurse is like, ‘they’ve got [vaginal speculums] with torches on them now,’ I was like, ‘Really? Clearly that was designed by a woman.’” (Jane, Riverland)

“My saving grace was it was an entire room full of women . . . I just felt so much safer in a room full of women . . . every other time it’s just been men telling me what I can and can’t do with my body.” (Faye, Ceduna)

#### Subtheme 2.3: No proactive care

Proactive care was missing in women’s experiences, which further eroded their shared understanding with health workers. Medical clinics had poor follow-up, as women recalled in the following quotations:

“I had a full blocking tube removed, and I just realised that I’ve never heard back from them.” (Cate, Ceduna).

“The only reason why I’ve seen doctors is because I’ve made appointments.” (Faye, Ceduna).

“Follow up care has been really, really hard. Once the midwives stopped coming, it was just basically impossible . . . you think if you had an actual, like if you really needed help, how do you get it without going to the hospital?” (Hana, Riverland).

Proactive care was strengthened when midwives attended medical appointments with women as “everyone’s in the loop” (Anne, Ceduna). Women in this study recalled shared understandings of health were built through their OB-GYN health appointments being prioritised at local health clinics. However, women recalled experiences of their health not being prioritised when booking appointments with clinic receptionists and in health clinics waiting rooms, as follows:

“If you rang her, she wouldn’t let you have a face-to-face. But then so she answered, I’d go, oh, sorry. Wrong number. I’d hang up and ring back two minutes later” (Diana, Ceduna).

“Last time I went for an appointment [with the obstetrician], there was three men that went in before me” (Billie, Ceduna).

Women in Ceduna recalled that their experience of proactive care drastically changed depending on their doctor. For example, one woman stated: “if it was with [doctor], she would’ve probably squeezed [me in]. She would’ve rung [me] herself” (Hana, Ceduna). In the Riverland, women reported doctors were poor in proactively checking information on their pregnancy record, a state government–funded hand-held antenatal record booklet that women are issued with at their first antenatal appointment.^45^

#### Subtheme 2.4: Disrupted care between local and city-based services

Travelling to Adelaide health services disrupted continuity of care, and women explored the difficulty in establishing shared understandings with city-based health professionals. As one woman recalled the differences of giving birth in Adelaide below,

“I felt like a cog in a machine. . . .it had been quite a scary experience, being flown to Adelaide. I didn’t know if I had a doctor, who I could talk to there. I just felt very much that I had to go with the flow. . .it wasn’t very personal, coming from the [Riverland] midwifery practice group, you know there’s eight midwives, three obstetricians, you’ve met them all, but [in Adelaide-based hospital], there’s hundreds of people. It felt impersonal, and I was sad” (Olive, Riverland).

Once returning to their local communities, many women reported poor quality follow-up, which is a standard practice in Australian public midwifery group settings.^46^ The following quote illustrates a woman’s difficulty re-joining regional health services after a traumatic delivery:

“But when I came home, I felt that everyone was just too scared. I didn’t have any midwifery care. . .the doctors in [the city hospital] had wanted the baby weighted every three days. . .no one weighed him. No one came to check on us . . . lucky that my partner’s home, because you know, I might have just gone and done something; who knows? I’ve got two other children, I’ve got a child who’s severely unwell, but no one can be bothered to check on us” (Faye, Ceduna).

### Theme 3: The language of choice, control, and consent

The language used in clinical encounters directly shapes women’s capacity for informed consent, choice, and control. Subtheme 3.1 shows health literacy as essential for agency and that unclear communication can significantly reduce autonomy. Subthemes 3.2, 3.3, and 3.4 illustrate how women respond to under-resourced systems by drawing on community support and their own understandings of health to maintain control. Together, these findings show that informed decision-making is an active negotiation within linguistic and systemic constraints.

#### Subtheme 3.1: Health literacy is intrinsic to informed consent

Women’s health literacy was identified as a necessary precursor to being able to give informed consent. Many women shared experiences where they felt health professionals had failed to affirm that they had the necessary health literacy to consent, leading to a loss of autonomy. The following quote illustrates this loss of agency experienced by women:

“[S]o then that doctor decided that [they] were going to break my waters, and I didn’t want [them] to, but [they] didn’t really explain what [they] was doing.” (Faye, Ceduna)

One woman recalled being given the choice between having a C-section that evening or the next morning, yet not appropriately informed on staff capabilities:

“They’re like, “Our C-section team is about to go home. So, you can either have one tonight or wait for the morning at 9am. [M]e being just turned 20, I was like, ‘Give me the C-section now,’ they could have just said; ‘Have the epidural, go to sleep for the night. They moved me into the surgery room, and [the doctor] was trying to put the spinal in for about half an hour.’” (Isla, Riverland).

Yet, women in this study were not passive when they experienced these obstacles. Instead, many actively made decisions according to their own health agency. The following two examples illustrate this:

“[W]hen I first got pregnant, I decided being in the health system, knowing what it’s like . . . [a]nd also knowing a lot of the staff members at the hospital, I decided that I wanted to give birth in Adelaide.” (Nola, Riverland)

“I’d rung the Loxton hospital and said, ‘If I’m going to get treated like I’m being stupid I’m not coming, but I am really not well.’” (Mila, Riverland)

#### Subtheme 3.2: Women’s choices and conceptualisations of health

Women in this study conceptualised women’s health as holistic to their mental, family, and social well-being, and made decisions accordingly:

“[I]t seemed like some of the earlier tests they do were just to see if there was something wrong with your baby, and so I also declined the test for Down Syndrome, or whatever, because I knew that I was going to keep the baby, so I’m like, that’s not worth the risk to test for something that a doctor’s just going to tell me to abort my baby anyway.” (Lana, Riverland)

Education backgrounds also shaped women’s health literacy and choices. As demonstrated in the quote below, a woman’s background as a mental health worker provided her with the literacy to identify mental health as part of OB-GYN health:

“It was ten days after birth, I’d been struggling, so I’d had low moods, crying. . .I had also been very proactive, because I’m a [mental health professional] through work, so I was very tuned in on how I was going. I’d organised to start seeing a perinatal psychologist before birth, so I had got a mental health plan . . . and yeah, I knew that it wasn’t right what I was . . . feeling.” (Olive, Riverland).

#### Subtheme 3.3: Communities advocate for women’s choices

Mothers were key relationships in passing down intergenerational knowledge of their own health experiences and choices. One grandmother-to-be stated: “I really wanted to be there for the labour and birth so that I could protect her from that overzealous monitoring” (Mila, Riverland). Fathers of children were viewed as equal partners in the birthing process, and choices were made to ensure partners were able to be present during births. Women shared the following quotes, which illustrate the importance of their support network during their pregnancy:

“I would’ve had a toddler by myself in Adelaide . . . so that’s what we opted for a chosen date so [my partner] could definitely be there.” (Anne, Ceduna)

“What if something is wrong, I don’t have my support person here . . . I’m trying to text him.” (Lana, Riverland)

One woman described how health professional’s advocacy shaped their access to critical care, whilst fostering two-way, reciprocal value and care in the community, as demonstrated below:

“The doctor who saved my vagina, I’d like to say, he had lost his wife six months beforehand and was a visiting specialist. . .my incredible midwife knew he was here – this was 2 o’clock in the morning, and she rang him and was like, ‘This girl needs you,’ and [the doctor] came in . . . he said to my midwife that was the first time he felt needed since his wife had died, and I was like, ‘I’m glad I did that for you, thank you for saving me.’” (Faye, Ceduna)

#### Subtheme 3.4: Under-resourcing undermines women’s control

Women recalled their male doctors denying their decisions for physiological birth and insisting on C-sections “because it was quick and easy, and they could just do it” (Faye, Ceduna). Women stated under-resourcing of local psychologists was voiced by male doctors as a reason to deny women referrals for psychologists. One woman recalled a conversation with her doctor as:

“[M]ental health is really hard in the Riverland because there are no doctors available. So, [he said] there’s no point putting in a wait list because you’re going to be on there for years. . .but I can give you this medication if you want.” (Kira, Riverland).

Under-resourcing of local services, together with health professionals’ poor health literacy, also negatively impacted women’s control. Women reported doctors sometimes used “Dr. Google” (Emma, Ceduna).

When visiting city hospital services, women living in Ceduna reported experiences where their doctors would deny their discharge to return to their remote townships. One woman recalled her doctor saying, “I’m not letting you go” and citing Ceduna’s closure of birthing services as the reason, despite her birthing plan already being in place and approved (Anne, Ceduna).

In Ceduna, women reported times that doctors discouraged vaginal birth after caesarean (VBAC) and did not inform women appropriately about their choices, as well as the associated risks and benefits. One woman felt her autonomy was ignored, stating: “I was severely bullied and basically made to feel like I was an idiot for even wanting to trust my body” (Faye, Ceduna). In circumstances of failed informed consent, choice and control, women identified the need for their knowledge to redefine dominant discourses on OB-GYN health. .

### Theme 4: Knowledge translation redefines OB-GYN health

Women’s OB-GYN experiences are often constrained within patriarchal, doctor-led, and bio-medical settings where institutional knowledge is frequently prioritised over their own. Subtheme 4.1 illustrates how these medical discourses normalise women’s pain and trauma, silencing their lived experiences. Conversely, subthemes 4.2 and 4.3 emphasise the importance of integrating medical and women’s knowledge systems, showing how women assert agency to question clinical diagnoses and advocate for midwife-led, non-intrusive care that affirms their authority over their holistic health.

#### Subtheme 4.1: Pain and risk normalised in women’s health care

Recalling their experiences, women stated health professionals commonly dismissed their experiences of health-related pain and trauma. Prior to the availability of a resident female doctor, who was known to always undertake Implanon™ procedures with pain medication, women reported the male doctors performed the procedure without pain medication: “whether you wanted to or not, it was go into the doctor’s office and do it whether it’s painful or anything; too bad” (Faye, Ceduna). Having painful vaginal births was also recalled in women’s experiences such as: “the birth itself was pretty traumatic, they tore me, I had a fourth-degree tear because they had to rip him out of me” (Faye, Ceduna).

Women stated post-partum depression and trauma were commonly dismissed by doctors as *baby blues* and “reinforced as quite a normal experience” (Olive, Riverland). A lack of early support for women who expressed concern about their postnatal emotions led to women developing severe postnatal depression and psychosis without being treated. Post-partum health checks focused on the child’s health, with women reporting their own mental health issues were ignored, as follows:

“[T]he doctor didn’t check me at all – checked [the baby] . . .it wasn’t until I was walking out that I’m like, oh hang on, you’re supposed to look at me . . . ‘oh okay, I’m forgotten about now.’” (Gina, Ceduna)

“[W]orst time in my life for my mental health.” (Lana, Riverland)

#### Subtheme 4.2: A desire for midwife-led and non-intrusive health care

A desire for midwifery-centred and non-intrusive health care was expressed by women as necessary to achieve holistic health. Doctors’ perception of pregnancy as inherently risky caused women’s choices to be ignored, as one woman shared:

“I wanted skin-to-skin . . . I didn’t want to pull my own baby out . . . and then they didn’t bring him back . . . then they brought him back all wrapped up really tight and they’re like, here, kiss your baby, which felt like – because there was a nurse who was taking photos, and it just felt so performative.” (Faye, Ceduna)

Women also recalled deciding not to do recommended medical tests that were seen as intrusive, with women expressing “I wasn’t ready for everyone to be all up in my bits” (Lana, Riverland). Women called for midwifery support and post-partum checks to extend beyond the 6-week standard. The following two quotes illustrate this:

“By myself with my baby, which was actually worse because I wasn’t coping, and I had another child to keep alive.” (Lana, Riverland)

“I want him passed straight from my belly to the boob, I don’t want him taken off to the table or anything. If you have to suction him out you can do that on my chest,” you know, blah, blah, blah, and [the doctor] agreed to all of that and used it to kind of educate everyone else in the room.” (Mila, Riverland)

#### Subtheme 4.3: The need to intertwine medical and women’s knowledge systems

Recalling their experiences, women in this study noted shared knowledge translation was necessary to bridge the gap between health professionals and women’s knowledge systems. Health professionals relying on medical knowledge alone, without listening to women’s bodily knowledge, led to women being given incorrect health diagnosis and care, as demonstrated in the following:

“[A]sked what happens to my baby if I go into labour, and they doctor at the time told me she wouldn’t be viable . . . I knew differently because my nieces were born at 24 weeks.” (Faye, Ceduna)

Women in this study voiced concern that many doctors did not view women’s own health literacy and bodily knowledge as important. As evidenced in the following quote, women are the authority on their own holistic health and have their own understandings of health, which health professionals should consider:

“[H]e was a bit like, ‘You don’t need a scan until 12 weeks.’ And I was like, ‘Okay. I’ve had a miscarriage previously. I just would like a dating scan . . . I knew I was about eight weeks then and [the doctor] was like, ‘Oh that’s unnecessary.’ . . . just didn’t give me a form.” (Anne, Ceduna)

Women were able to attain appropriate health care by two-way knowledge exchange between women and their doctors, as demonstrated in the following quotation:

“The [doctor] I really liked best (. . .) that I saw giving birth (. . .) said ‘You know, women have been doing this for a long time. This is what your body can do’ (. . .) and he was a bit more hands off, which is why I really liked him.” (Lana, Renmark)

Shared knowledge exchange also strengthened women’s health literacy, positively reinforcing their access to health care and their agency.

## Discussion

Our study found non-Indigenous women living in rural and remote areas of South Australia were disadvantaged by poor access to OB-GYN health care and services, heightening their risk of traumatic health experiences and outcomes. By applying a feminist post-structuralist lens, our article follows previous literature to phenomenologically explore experiences of OB-GYN health care through a critical feminist lens.^47–49^ This highlights the way women navigate access to health care with agency, whilst conceptualising this care as embedded in existing power and knowledge inequity.^49^ To the authors knowledge, however, this feminist post-structuralist analysis in the context of rural and remote OB-GYN health systems is unique in Australia. This lens is an important contribution for rural and remote research. It exposes how gendered and geographical power structures are systemically embedded in OB-GYN services, sculpting and upholding bio-medical knowledge systems and thereby undermining women’s embodied health, knowledge, and agency. Only by unveiling covert power structures is this lens able to foreground rural and remote women’s distinct and embodied knowledge to inform OB-GYN services and tackle their structural marginalisation and health disparity.

Systemically, our article finds that gendered inequity interacts with, and is reinforced by, severe under-resourcing of rural and remote health care, and medicalised *othering* of women’s health, choice, pain, and knowledge. Our research findings demonstrate this inequity mitigates women’s access to health care and, thereby, exists in traction to women’s exertion of health agency.

### Power inequities within women’s health

In some very profound ways, women in this study identified how power inequity in accessing health care was specific to their experiences of medical misogyny and being a woman within a health system designed by, and for, men.^50,51^ Gender bias has been documented within Australian,^52,53^ and international,^54^ healthcare services, preventing women’s access to appropriate diagnosis, and care. Gender bias is not only exerted through individual healthcare professionals; rather, women’s experiences illustrated that it exists systemically in policies and practices^48^ reflective in pervasive medical research being conducted on men and thereby excluding women’s experiences as the pathological “other.”^54,55^ At Australia’s 2024 National Women’s Health Summit, two in three women reported experiencing gender bias when accessing health care.^52^

Women’s experiences of pain were embedded in their experiences of accessing health care, yet systematically dismissed.^22,50,53^ As found in international studies, women reported health professionals provided inadequate pain examination and management during post-partum,^50,51^ and chronic pelvis pain,^56^ appointments. Chronic pain impacts high proportions of women in Australia; however, women are less likely to receive diagnosis or treatment, reflective of systemic biomedical biases that normalise the aetiology of women’s pain.^53^ In rural and remote areas of Australia, a shortage of multi-disciplinary health professionals, such as psychologists and physiotherapists, intensifies power inequities in accessing care for pain.^53^ Despite this, minimal studies focus on rural and remote women’s experiences of pain during women’s health care.

Increasing literature reports women’s experiences of birth as traumatic, violating, and dehumanising.^22,48,57^ Though focused in urban areas, this literature mirrors the findings reported herein, which found non-consensual vaginal examinations, coerced caesarean sections, and negative interactions with healthcare professionals force women to experience trauma.^22,48,57^ Furthermore, bio-medical knowledge biases reinforce health professionals’ authority over women’s bodies.^22^ Denial of bodily control during labour increases women’s risk of developing post-partum depression, psychosis, and PTSD,^22,57^ all of which were reported by women in our study.

Our findings based in South Australia mirror other Australian states, such as Dietsch et al.’s findings in New South Wales, where women who travelled from their rural and remote townships experienced power inequity in the form of uncompassionate care from their midwives.^58^ Building upon these findings, which are largely obstetric focused, our study has established that power inequity also occurs in gynaecological healthcare services. To resolve these power and health inequities, studies recommend trauma-informed care, where health professionals prioritise understanding women’s experiences and address these understandings during their care.^56,59,60^

Our study’s finding of health professionals normalising poor mental health outcomes as “baby-blues,” and the stigma women experienced in reporting poor post-partum mental health, has not been identified in other Australian literature. This is an area that needs further research and policy attention.

### Under-resourcing in isolated communities

Women in this study navigated severe under-resourcing of local female health staff and services, as in other literature on women living in rural and remote areas.^9,61,62^ In our study, we uniquely found that the pervasive lack of resources in rural and remote general health led to women subjectively experiencing their health needs as being *othered* and not prioritised.^62^

As in our study, Mathews et al. explored women’s pregnancy experiences in the Riverland of South Australia, yet differ in solely focusing on women with high-risk pregnancies.^62^ Their findings, similar to other literature,^9,61^ revealed women were burdened with heightened socio-financial, emotional, and logistical stresses, whilst being isolated from community networks, and feeling that city health professionals lacked understanding of their burden of rurality when re-located to city-based care.^62^ Additionally, our study found that rural and remote women’s travel requirements are intensified by local under-resourcing of mental health, lactation, and physiotherapists services.^9,61^

To minimise rural and remote barriers to OB-GYN services, tele-health may offer women living rurally and remotely access to health care.^62,63^ Even when accessing services locally, inadequate *bulkbilling* (a service that charges health care to Australia’s universal health insurance scheme),^9^ and a lack of choice between local bulk billing health providers, minimised women’s access to health care in our study cohort. To overcome this, our research recommends increased funding for midwifery care pathways and free perinatal care, as this has been found to reduce women’s OB-GYN-related financial and mental stress.^61^ Furthermore, our findings support training initiatives for general practice nurses to undertake cervical screening tests in rural and remote areas, potentially alleviating doctor burn-out, staffing shortages, and increasing available appointments.^64^

By exploring women’s experiences living remotely, our research is able to contribute to rural based studies,^61,62^ establishing that remoteness intensifies the risk of socio-financial and resource inequity. Herein, our study responds to a paucity in literature on how varying degrees of remoteness, under-resourcing, and having a high-risk pregnancy intersect and intensify health inequity, whilst undermining women’s health autonomy.

### Women’s agency to facilitate their own health

To access women-centred health care, whilst navigating systemic biases against their knowledge and choices, women in our study cohort exerted their agency to facilitate their own health, reiterating findings reported elsewhere.^9,26–28,65,66^ Global literature has found women-enacting agency increases their access to skilled antenatal and postnatal care^27^ and reproductive care.^39^ Our findings are significant in identifying women’s health agency in rural and remote areas. Determinants of agency have been shown to include having higher levels of education, being older than 30, involved socio-economic decision-making, and having partners in attendance at healthcare appointments.^39^ Similarly, our cohort experienced a loss of control when COVID-19 restrictions prevented their partners from attending obstetric appointments, as found in international literature.^67^ We also found lower agency amongst first-time mothers and women with less experience navigating the health system, as described in other cohorts.^39^

In our study, women’s access to VBACs and caesareans with skin-to-skin contact, as well as navigating a bio-medical preference for caesareans, was a large aspect of their need to exert agency. Metropolitan-centric literature has explored women’s experiences of health professionals using coercive language to legitimise their control and dissuade women from accessing VBAC’s.^48,68^ Though not applied with a lens of agency, women have navigated this barrier by accessing information online, social networks, and attempted to find supportive health professionals.^68^ Herein, women were not static in their access of health care, but rather sought out woman-centred care. Research has shown birth planning enables women’s agency during caesarean sections and their access to immediate post-birth skin-to-skin care.^69^

Amongst our cohort, socio-health relationships strengthened women’s agency in decision-making. This is consistent with research reported elsewhere.^9,39,61,62^ Continuity of care has been found to increase rural women’s agency and their involvement in decision-making, whilst reducing their difficulty navigating the health system between city and local-based care.^61,66^ Access to rural Midwifery Group Practice (MGP) programmes, a model of maternity care that pairs women with a regular midwife to ensure continuity of care, has improved women’s agency in rural Australia by facilitating midwifery-care that prioritised listening to, respecting, and involving women in decision-making,^66^ as found in our study. Furthermore, MGP minimised negative impacts that forced closures of birthing services has on women’s agency in rural areas.^66^ The need for ongoing communication with local health professionals has strengthened women’s sense of control in other studies.^62^ As in our research, poor relationships with health professionals minimises women’s capacity to advocate for oneself and disclose domestic violence.^61^ This highlights the importance of women having access to health networks and literacy to support their exertion of agency.

### Health literacy

All women in our study highlighted health literacy, and their access to information, as a necessary precursor to safe women’s health care.^65,70^ Having access to, and the skills to navigate, online health information enables women in rural and remote communities to access perinatal depression resources when otherwise unavailable.^61^ Health professionals’ advice, speaking with their family and friends, and conducting their own online research, were information sources utilised by our cohort, in line with previous research.^28,65,71^ The desire for holistic and individualistic health care drives women to actively seek relevant information from trusted sources and be proactive participants in their own health journey, to feeling heard, and having their opinions valued.^65^ Women desired for information to be delivered with individualistic consideration of their needs.^71^ However, research has also shown women from regional areas prefer to access information regarding their gynaecological care outside community relationships.^71^ This may be indicative of the underlying privacy concerns raised amongst the women in our study. Ensuring health information is provided in ways that meet women’s needs in rural and remote locations is clearly necessary. Co-designing gynaecological health information with women and training for health professionals have been recommended approaches to improving access and health literacy.^71^

### Strengths, limitations, and future research

Previous research has primarily focused on rural women’s experiences and lack of agency; however, herein we have addressed this persistent research gap by including women living in remote locations, and their experiences of exerting agency to access women’s health care. This article reflects the lived experiences of women in our study, demonstrating their experiences of inadequate consent and pain, whilst accessing OB-GYN care, and to varying degrees depending on their remoteness. Our study fills a gap in addressing women’s experiences enacting agency to access gynaecological care in remote Australia.

Another strength of this research is our iterative application of a feminist post-structuralist framework to understand these women’s lived experiences of power, knowledge, and health inequity. This has demonstrated how women’s subjectivity and agency challenged health system failures, reconfirming their embodiment of health. The consideration of different theoretical frameworks in our future research will be dependent on the women’s experiences and socio-cultural backgrounds. Other studies have applied this framework to understand women’s health during menopause,^28^ and pregnancy loss,^72^ yet to the authors knowledge, this article is the first to utilise a feminist post-structuralist framework to explore women’s rural and remote access to health care in the Australian context.

A potential limitation of this study is its focus on data from non-Indigenous women; however, the authors recognise the importance of First Nations women’s experiences living across rural and remote locations. As such, we have partnered with First Nations researchers to undertake Indigenous yarning methodology and co-led research with Aboriginal women on Aboriginal women’s health across remote parts of South Australia. This research will be reported elsewhere. Future research could examine the similarities and differences in the experiences of non-Indigenous and First Nations women’s access to health.

Another potential limitation was most of our cohort were younger women who had young children, that is, pre-menopausal. Whilst the authors sought to recruit older women in the study cohort, this was a difficult age range to reach. However, the authors acknowledge this is an important demographic in understanding how women access care, and future research should be conducted with peri- or post-menopausal women to understand their unique experiences. In addition, our interview guide focused on accessing obstetric and/or gynaecological services and whilst contraception and family planning options were discussed as part of our conversations, access to abortion was not raised by any of the women in this cohort. The authors recognise access to abortion care is a highly important issue in rural and remote areas. The authors recommend further research focus on access to abortion care in rural and remote Australia. Finally, future research should include interviews with key women’s health professionals to understand their experiences navigating the rural and remote healthcare system.

## Conclusion

Our study describes the OB-GYN healthcare experiences of non-Indigenous women living in rural and remote South Australia. In summary, women experienced poor access to OB-GYN health care and services and often had traumatic health experiences and outcomes. Women acknowledged much of this was due to severe under-resourcing of rural and remote health services and overcame these inequities by exerting their agency and autonomy to seek out the health information and care they required. As a result of our findings, we recommend policy and strategy development to support women’s agency in their access to OB-GYN care to reduce women’s experiences of morbidity and mortality. Policies and strategies must address women’s health care in a holistic way, whilst prioritising women living in remote locations, having lower socio-economic backgrounds, or underlying morbidities which increase health risks and inequity.

## Supplemental Material

sj-pdf-1-whe-10.1177_17455057261444883 – Supplemental material for Non-Indigenous women’s experiences of obstetric and gynaecological inequity in rural and remote South Australia: A feminist post-structuralist analysis Supplemental material, sj-pdf-1-whe-10.1177_17455057261444883 for Non-Indigenous women’s experiences of obstetric and gynaecological inequity in rural and remote South Australia: A feminist post-structuralist analysis by Laura Nolan, Elizabeth Newnham and Jacqueline H Stephens in Women's Health
