# Supplementary material for: Non-Indigenous women’s experiences of obstetric and gynaecological inequity in rural and remote South Australia: A feminist post-structuralist analysis
Source: Womens Health (Lond). 2026 Jun 9;22:17455057261444883. doi: 10.1177/17455057261444883 (PMC13254423; doi:10.1177/17455057261444883)
Supplement: sj-pdf-1-whe-10.1177_17455057261444883 – Supplemental material for Non-Indigenous women’s experiences of obstetric and gynaecological inequity in rural and remote South Australia: A feminist post-structuralist analysis [file sj-pdf-1-whe-10.1177_17455057261444883.pdf]

SUPPLEMENTARY FILE

Contents

1. Standards for Reporting Qualitative Research (SRQR) ... 2
2. Interview Guide ... 4

1. Standards for Reporting Qualitative Research (SRQR)

More information available at: http://www.equator-network.org/reporting-guidelines/srqr/

**Title and abstract**	
**Title** - Concise description of the nature and topic of the study Identifying the study as qualitative or indicating the approach (e.g., ethnography, grounded theory) or data collection methods (e.g., interview, focus group) is recommended	Page 1
**Abstract** - Summary of key elements of the study using the abstract format of the intended publication; typically includes background, purpose, methods, results, and conclusions	Page 2
**Introduction**	
**Problem formulation** - Description and significance of the problem/phenomenon studied; review of relevant theory and empirical work; problem statement	Page 3
**Purpose or research questio**n - Purpose of the study and specific objectives or questions	Page 4
**Methods**	
**Qualitative approach and research paradigm** - Qualitative approach (e.g., ethnography, grounded theory, case study, phenomenology, narrative research) and guiding theory if appropriate; identifying the research paradigm (e.g., postpositivist, constructivist/ interpretivist) is also recommended; rationale**	Pages 4-5
**Researcher characteristics and reflexivity** - Researchers' characteristics that may influence the research, including personal attributes, qualifications/experience, relationship with participants, assumptions, and/or presuppositions; potential or actual interaction between researchers' characteristics and the research questions, approach, methods, results, and/or transferability	Page 5
**Context** - Setting/site and salient contextual factors; rationale**	Page 6
**Sampling strategy** - How and why research participants, documents, or events were selected; criteria for deciding when no further sampling was necessary (e.g., sampling saturation); rationale**	Page 7
**Ethical issues pertaining to human subjects** - Documentation of approval by an appropriate ethics review board and participant consent, or explanation for lack thereof; other confidentiality and data security issues	Page 6
**Data collection methods** - Types of data collected; details of data collection procedures including (as appropriate) start and stop dates of data collection and analysis, iterative process, triangulation of sources/methods, and modification of procedures in response to evolving study findings; rationale**	Page 7
**Data collection instruments and technologies** - Description of instruments (e.g., interview guides, questionnaires) and devices (e.g., audio recorders) used for data collection; if/how the instrument(s) changed over the course of the study	Page 7, Supplementary File
**Units of study** - Number and relevant characteristics of participants, documents, or events included in the study; level of participation (could be reported in results)	Page 7

**Data processing** - Methods for processing data prior to and during analysis, including transcription, data entry, data management and security, verification of data integrity, data coding, and anonymization/de-identification of excerpts	Pages 7-8
**Data analysis** - Process by which inferences, themes, etc., were identified and developed, including the researchers involved in data analysis; usually references a specific paradigm or approach; rationale**	Page 8
**Techniques to enhance trustworthiness** - Techniques to enhance trustworthiness and credibility of data analysis (e.g., member checking, audit trail, triangulation); rationale**	Page 7
**Results/findings**	
**Synthesis and interpretation** - Main findings (e.g., interpretations, inferences, and themes); might include development of a theory or model, or integration with prior research or theory	Pages 8-21, Figure 1
**Links to empirical data** - Evidence (e.g., quotes, field notes, text excerpts, photographs) to substantiate analytic findings	Pages 8-21
**Discussion**	
**Integration with prior work, implications, transferability, and contribution(s) to the field -** Short summary of main findings; explanation of how findings and conclusions connect to, support, elaborate on, or challenge conclusions of earlier scholarship; discussion of scope of application/generalizability; identification of unique contribution(s) to scholarship in a discipline or field	Pages 22-26
**Limitations** - Trustworthiness and limitations of findings	Pages 26-27
**Other**	
**Conflicts of interest** - Potential sources of influence or perceived influence on study conduct and conclusions; how these were managed	Declarations
**Funding** - Sources of funding and other support; role of funders in data collection, interpretation, and reporting	Declarations

2. Interview Guide

GUIDING QUESTIONS FOR THE FACILITATOR

These are guiding questions, only. Topics raised by participants should also be explored.

Pregnancy health related questions

1. What was your experience of the pregnancy or maternal care you received? What was good? What was not good? What could have been improved?

2. Did anyone experience any problems accessing pregnancy healthcare (from any health professional or health setting)? What were these problems? Were there any language barriers? How long did you have to wait to get an appointment?

3. What was your experience communicating with the health care provider? Did you feel that your needs were heard, and that you received culturally appropriate care?

4. If you could say anything to the health professional (e.g. Obstetrician/GPO/Midwife/Other) about the care they provided to you, what would you say?

5. How far did you have to travel to receive care? How did that impact on your family and other responsibilities?

6. How might health professionals improve pregnancy health services to women in your area?

7. Have you used telehealth services (i.e. health services over the phone or a video call) in the past 18 months? If so, what has been your experience of using telehealth?

8. Do you think COVID-19 had an impact on the care you received? If so, what was good? What was not good?

Gynaecology related questions

1. What was your experience of the women's health (gynaecological) care you received? What was good? What was not good? What could have been improved?

2. Did anyone experience any problems accessing care for a women's health condition (from any health professional or health setting)? What were these problems? Were there any language barriers? How long did you have to wait to get an appointment?

3. What was your experience communicating with the health care provider? Did you feel that that your needs were taken into consideration, and that you received culturally appropriate care?

4. If you could say anything to the health professional about the care they provided to you, what would you say?

5. How far did you have to travel to receive care? How did that impact on your family and other responsibilities?

6. How might health professionals improve women's health services to women in your area?

7. Have you used telehealth (i.e. health services over the phone or a video call) services in the past 18 months? If so, what has been your experience of using telehealth?

8. Do you think COVID-19 had an impact on the care you received? If so, what was good? What was not good?
